# The natural algorithmic approach of mixed trigonometric-polynomial problems

**DOI:** 10.1186/s13660-017-1392-1

**Published:** 2017-05-18

**Authors:** Tatjana Lutovac, Branko Malešević, Cristinel Mortici

**Affiliations:** 1 Faculty of Electrical Engineering, University of Belgrade, Bulevar kralja Aleksandra 73, Belgrade, 11000 Serbia; 2 Valahia University of Târgovişte, Bd. Unirii 18, Târgovişte, 130082 Romania; 3 Academy of Romanian Scientists, Splaiul Independenţei 54, Bucharest, 050094 Romania; 4 University Politehnica of Bucharest, Splaiul Independenţei 313, Bucharest, 060042 Romania

**Keywords:** 41A10, 26D05, 68T15, 12L05, 41A58, mixed trigonometric-polynomial functions, Taylor series, approximations, inequalities, algorithms, automated theorem proving

## Abstract

The aim of this paper is to present a new algorithm for proving mixed trigonometric-polynomial inequalities of the form

$$\sum_{i=1}^{n}\alpha _{i}x^{p_{i}} \cos ^{q_{i}} x\sin ^{r_{i}} x>0 $$

by reducing them to polynomial inequalities. Finally, we show the great applicability of this algorithm and, as an example, we use it to analyze some new rational (Padé) approximations of the function cos^2^ *x* and to improve a class of inequalities by Yang. The results of our analysis could be implemented by means of an automated proof assistant, so our work is a contribution to the library of automatic support tools for proving various analytic inequalities.

## Introduction and motivation

In this paper, we propose a general computational method for reducing some inequalities involving trigonometric functions to the corresponding polynomial inequalities. Our work has been motivated by many papers [1–13] recently published in this area. As an example, we mention the work of Mortici [3] who extended Wilker-Cusa-Huygens inequalities using the method he called *the natural approach method*. This method consists in comparing and replacing sin*x* and cos*x* by their corresponding Taylor polynomials as follows:

$$\begin{aligned}& \sum_{i=0}^{2s+1} \frac{(-1)^{i}x^{2i+1}}{(2i+1)!} < \sin x < \sum_{i=0}^{2s} \frac{(-1)^{i}x^{2i+1}}{(2i+1)!}, \\& \sum_{i=0}^{2k+1}\frac{(-1)^{i}x^{2i}}{(2i)!} < \cos x < \sum_{i=0}^{2k} \frac{(-1)^{i}x^{2i}}{(2i)!} \end{aligned}$$

for every integer $s, k \in \mathbb {N}_{0}$ and $x \in (0,\pi/2 )$.

In this way, complicated trigonometric expressions can be reduced to polynomial or rational expressions, which can be, at least theoretically, easier studied (this can be done using some software for symbolic computation, such as Maple).

For example, Mortici in [3] (Theorem 1) proved the following inequality:

$$\cos x - \biggl( \frac{\sin x}{x} \biggr)^{ 3} > - \frac{x^{4}}{15}, \quad x \in \biggl(0, \frac{\pi }{2} \biggr), $$

by intercalating the following Taylor polynomials:

$$\cos x - \biggl(\frac{\sin x}{x} \biggr)^{ 3} + \frac{x^{4}}{15} > 1 - \frac{x^{2}}{2!}+\frac{x^{4}}{4!}-\frac{x^{6}}{6!} - \biggl( \frac{ x - \frac{x^{3}}{3!} + \frac{x^{5}}{5!}}{x} \biggr)^{ 3 } + \frac{x^{4}}{15} = \frac{x^{6}R (x^{2} )}{1{,}728{,}000}, $$

where $R ( t ) =20{,}000-1{,}560t+60t^{2}-t^{3}$.

Let $\delta _{1} \leq 0 \leq \delta _{2}$, with $\delta _{1} < \delta _{2}$. Recall that a function defined by the formula

1 $$ f(x)=\sum_{i=1}^{n}\alpha _{i}x^{p_{i}} \cos ^{q_{i}} x\sin ^{r_{i}} x, \quad x \in (\delta _{1},\delta _{2}), $$

is named a mixed trigonometric-polynomial function, denoted in the sequel by an MTP function [8, 14]. Here, $\alpha _{i} \in \mathbb{R} \setminus \{0\}$, $p_{i}, q_{i}, r_{i} \in \mathbb{N}_{0}$, $n \in \mathbb{N}$. Moreover, an inequality of the form $f(x) > 0$ is called a mixed trigonometric-polynomial inequality (MTP inequality).

MTP functions currently appear in the monographs on the theory of analytical inequalities [15, 16] and [5], while concrete MTP inequalities are employed in numerous engineering problems (see, e.g., [17, 18]). A large class of inequalities arising from different branches of science can be reduced to MTP inequalities.

It is notable that many of the above-mentioned analyses and treatments of MTP inequalities are all rather sophisticated and involve complex transformations and estimations. Almost all approaches are designed for ’pen and paper analysis’ and many of them are ripe for automation, being formally defined in precise detail, and yet somewhat overwhelming for humans.

Notwithstanding, the development of formal methods and procedures for automated generation of proofs of analytical inequalities remains a challenging and important task of artificial intelligence and automated reasoning [19, 20].

The aim of this paper is to develop a new algorithm, based on the natural approach method, for proving MTP inequalities by reducing to polynomial inequalities.

Although transformation based on the natural approach method has been made by several researchers in their isolated studies, a unified approach has not been given yet. Moreover, it is interesting to note that just trigonometric expressions involving odd powers of cos*x* were studied, as the natural approach method cannot be directly applicable for the function cos^2^ *x* over the entire interval $(0, \pi/2)$. Our aim is to extend and formalize the ideas of the natural approach method for a wider class of trigonometric inequalities, including also those containing even powers of cos*x*, with no further restrictions.

Notice the logical-hardness general problem under consideration. According to Wang [21], for every function *G* defined by arithmetic operations and a composition over polynomials and sine functions of the form $\sin \pi x$, there is a real number *r* such that the problem $G(r)=0$ is undecidable (see [22]). In 2003, Laczkovich [23] proved that this result can be derived if the function *G* is defined in terms of the functions $x,\sin x$ and $\sin (x\sin x^{n})$, $n=1,2,\ldots $ (without involving *π*). On the other hand, several algorithms [24, 25] and [26] have been developed to determine the sign and the real zeroes of a given polynomial, so that such problems can be considered decidable (see also [22, 27]).

Let us denote by

$$T_{n}^{\phi, a}(x) = \sum_{k=0}^{n} \frac{\phi^{(k)}(a)}{k!}(x-a)^{k} $$

the Taylor polynomial of *n*th degree associated with the function *ϕ* at a point *a*. Here, $\overline{T}_{n}^{\phi ,a}(x)$ and $\underline{T}_{n}^{\phi ,a}(x)$ represent the Taylor polynomial of *n*th degree associated with the function *ϕ* at a point *a*, in the case $T_{n}^{\phi,a}(x) \geq \phi(x)$, respectively $T_{n}^{\phi, a}(x) \leq \phi(x)$, for every $x\in ( a,b )$. We will call $\overline{T}_{n}^{\phi ,a}(x)$ and $\underline{T}_{n}^{\phi ,a}(x) $ an upward and a downward approximation of *ϕ* on $(a,b )$, respectively.

We present a new algorithm for approximating a given MTP function $f(x)$ by a polynomial function $P(x)$ such that

2 $$ f(x)>P(x), $$

using the upward and downward Taylor approximations $\underline{T}_{ n}^{\sin ,0}{(x)}$, $\overline{T}_{n}^{ \sin ,0}{(x)}$, $\underline{T}_{ n}^{\cos ,0}{(x)}$, $\overline{T}_{n}^{ \cos, 0}{(x)}$.

## The natural approach method and the associated algorithm

The following two lemmas [8] related to the Taylor polynomials associated with sine and cosine functions will be of great help in our study.

### Lemma 1

*Let* $T_{n}(x) = \sum_{i=0}^{(n-1)/2} \frac{(-1)^{i}x^{2i+1}}{(2i+1)!}$.

(i) *If* $n=4s+1$, *with* $s\in \mathbb {N}_{0}$, *then*
  3 $$ T_{n}(x)\geq T_{n+4}(x)\geq \sin x \quad \textit{for every } 0 \leq x\leq \sqrt{(n+3) (n+4)}; $$
  *and*
  4 $$ T_{n}(x)\leq T_{n+4}(x)\leq \sin x \quad \textit{for every } {-}\sqrt{(n+3) (n+4)}\leq x\leq 0. $$
(ii) *If* $n=4s+3$, *with* $s\in \mathbb {N}_{0}$, *then*
  5 $$ T_{n}(x)\leq T_{n+4}(x)\leq \sin x \quad \textit{for every } 0 \leq x\leq \sqrt{(n+3) (n+4)}; $$
  *and*
  6 $$ T_{n}(x)\geq T_{n+4}(x)\geq \sin x \quad \textit{for every }{-}\sqrt{(n+3) (n+4)}\leq x\leq 0. $$

### Lemma 2

*Let* $T_{n}(x)= \sum_{i=0}^{n/2} \frac{(-1)^{i}x^{2i}}{(2i)!}$.

(i) *If* $n=4k$, *with* $k\in \mathbb{N}_{0}$, *then*
  7 $$\begin{aligned}& T_{n}(x)\geq T_{n+4}(x)\geq \cos x \\ & \quad\textit{for every } {-}\sqrt{(n + 3) (n + 4)} \leq x \leq \sqrt{(n + 3) (n + 4)}. \end{aligned}$$
(ii) *If* $n=4k+2$, *with* $k\in \mathbb{N}_{0}$, *then*
  8 $$\begin{aligned}& T_{n}(x)\leq T_{n+4}(x)\leq \cos x \\& \quad \textit{for every } {-}\sqrt{(n + 3) (n + 4)} \leq x \leq \sqrt{(n + 3) (n + 4)}. \end{aligned}$$

According to Lemmas 1-2, the upper bounds of the approximation intervals of the functions sin*x* and cos*x* are $\varepsilon _{1}= \sqrt{(n_{1}+3)(n_{1}+4)}$ and $\varepsilon _{2}=\sqrt{(n_{2}+3)(n_{2}+4)}$, respectively. As $\varepsilon_{1}> \frac{\pi}{2}$ and $\varepsilon _{2}> \frac{\pi}{2}$, the results of these lemmas are valid, in particular, in the entire interval $( 0, \frac{\pi}{2} )$.

### Lemma 3

1. *Let* $n\in \mathbb {N}$ *and* $x\in ( 0,\frac{\pi}{2} ) $. *Then*
  $$T_{n}^{ \sin ,0}(x) \geq 0. $$
2. *Let* $s\in \mathbb {N}_{0}$, $p\in \mathbb {N}$ *and* $x\in ( 0, \frac{\pi}{2} )$. *Then*
  $$\bigl( \underline{T}_{4s+3}^{ \sin ,0}(x) \bigr) ^{p}\leq \sin ^{p}{ x}\leq \bigl( \overline{T}_{4s+1}^{ \sin ,0}(x) \bigr) ^{p}. $$

### Lemma 4

*Let* $k\in \mathbb{N}_{0}$, $p\in \mathbb{N}$ *and* $x\in ( 0, \frac{\pi}{2} ) $. *Then*

$$\cos ^{ p}{ x\leq } \bigl( \overline{T}_{4k}^{ \cos ,0}(x) \bigr) ^{p}. $$

In contrast to the function sin*x* and its downward Taylor approximations, in the interval $( 0, \frac{\pi}{2} ) $ the function cos*x* and the downward Taylor approximations $\underline{T}_{ 4k+2}^{ \cos ,0}(x)=\sum_{i=0}^{2k+1}{ \frac{(-1)^{i}x^{2i}}{(2i)!}}, k \in \mathbb {N}_{0}$, require special attention as there is no downward Taylor approximation $\underline{T}_{ 4k+2}^{ \cos ,0}(x)$ such that $\cos ^{2}{ x} \geq ( \underline{T}_{ 4k+2}^{ \cos ,0}(x) ) ^{2}$ for every $x\in ( 0, \frac{\pi}{2} )$.

We present the following results related to the problem with downward Taylor approximations of the cosine function.

### Proposition 5

1. *For every* $k\in \mathbb{N}_{0} $, *the downward Taylor approximation* $\underline{T}_{ 4k+2}^{ \cos ,0}(x)$ *is a strictly decreasing function on* $( 0, \frac{\pi}{2} ) $.
2. *For every* $k\in \mathbb{N}_{0} $, *there exists unique* $c_{k}\in ( 0, \frac{\pi}{2} ) $ *such that* $\underline{T}_{ 4k+2}^{ \cos,0}(c_{k})=0$.
3. *The sequence* $(c_{k} ) _{k\in\mathbb{N}_{0}}$, *with* $c_{0}=\sqrt{2}$, *is strictly increasing and* $\lim_{k\rightarrow +\infty }{c_{k}}=\frac{\pi }{2}$.
4. *For every* $k\in \mathbb {N}_{0}$, *there exists* $d_{k}\in ( c_{k}, \frac{\pi}{2} )$ *such that* $\cos {d_{k}} = \vert \underline{T}_{ 4k+2}^{ \cos ,0}(d_{k}) \vert $.
5. *The sequence* $( d_{k} ) _{k\in \mathbb{N}_{0}}$ *is strictly increasing and* $\lim_{k\rightarrow +\infty }{ d_{k}}=\frac{\pi }{2}$.

### Proof

(1) The function $\underline{T}_{ 4k+2}^{\cos ,0}(x)$ is strictly decreasing on $( 0,\frac{\pi}{2} )$ since, according to Lemma 1, $( \underline{T}_{ 4k+2}^{ \cos ,0}(x) ) ^{\prime} = -\overline{T}_{4k+1}^{ \sin ,0} (x) \leq 0$.

(2) The existence of $c_{k}$ follows from the fact that $\underline{T}_{ 4k+2}^{ \cos ,0}(0) = 1 > 0$ and $\underline{T}_{ 4k+2}^{ \cos ,0} ( \frac{\pi}{2} ) < \cos { ( \frac{\pi}{2} )} = 0$.

(3) The monotonicity of the sequence $(c_{k})_{k\in \mathbb{N}_{0}}$ is a result of the monotonicity of $\underline{T}_{ 4k+2}^{ \cos ,0}(x)$ and Lemma 2(ii).

The convergence of the sequence $(T_{n}^{ \cos ,0}(x))_{n\in \mathbb{N}}$ implies the convergence of the sequence $(c_{k})_{k\in \mathbb {N}_{0}}$ to $\frac{\pi}{2}$.

(4) The function $\vert \underline{T}^{ \cos ,0}_{4k+2}(x) \vert $ is decreasing on $(0,c_{k})$ and increasing on $(c_{k},\frac{\pi}{2} )$. Based on Lemma 2(ii), it follows that there exists $d_{k} \in ( c_{k}, \frac{\pi}{2} )$ such that $\cos{d_{k}} = \vert \underline{T}^{ \cos ,0}_{ 4k+2}(d_{k}) \vert $.

(5) This statement is a consequence of the monotonicity of the sequence $( c_{k})_{k\in \mathbb{N}_{0}}$ and the increasing monotonicity of the function $\vert \underline{T}_{4k+2}^{ \cos,0}(x) \vert $ on $(c_{k},\frac{\pi}{2} )$. □

### Corollary 6

*Let* $k\in \mathbb {N}_{0}$ *and* $p\in \mathbb {N}$. *Then*

1. $\cos ^{2p}{ x}> ( \underline{T}_{4k+2}^{ \cos ,0}(x) )^{2p}$ *for every* $x\in (0,d_{k})$;
2. $\cos ^{2p}{ x}< ( \underline{T}_{4k+2}^{ \cos ,0}(x) ) ^{2p}$ *for every* $x\in (d_{k}, \frac{\pi}{2} )$.

Based on the above results, we have the following.

### Corollary 7

*Let* $k\in \mathbb {N}_{0}$ *and* $p\in \mathbb {N}$. *Then* $\underline{T}_{ 4k+2}^{ \cos ,0}(x)$ *is not a downward approximation of the MTP function* $\cos^{2p}{x}$ *on* $(d_{k}, \frac{\pi}{2} )$.

In order to ensure the correctness of the algorithm [27, 28] we will develop next in the sequel, the following problem needs to be considered.

### Problem

For given $\delta \in (0, \frac{\pi}{2} )$ and $\mathcal{I} \subseteq (0, \frac{\pi}{2} )$, find $\widehat{k} \in \mathbb{N}_{0}$ such that for all $k \in \mathbb{N}_{0}$, $k \geq \widehat{k}$ and $x \in \mathcal{I}$

9 $$ \cos ^{2}{ x}\geq \bigl( \underline{T}_{ 4k+2}^{ \cos,0}(x) \bigr) ^{2}. $$

### Remark

If cos*x* appears in odd powers only in the given MTP function $f(x)$, we take $\widehat{k}=0$.

One of the methods to solve the problem of downward approximation of the function $\cos ^{2p}{ x}, p\in\mathbb{N}$ is the method of multiple angles developed in [8]. All degrees of the functions sin*x* and cos*x* are eliminated from the given MTP function $f(x)$ through conversion into multiple-angle expressions. This removes all even degrees of the function cos*x*, but then sine and cosine functions appear in the form $\sin \boldsymbol{\kappa} x$ or $\cos \boldsymbol{\kappa} x$, where $\boldsymbol{\kappa} x \in ( 0, \boldsymbol{\kappa} \frac{\pi}{2} )$ and $\boldsymbol{\kappa} \in \mathbb{N}$. In this case, in order to use the results of Lemmas 1-2, we are forced to choose large enough values of $k \in \mathbb{N}_{0}$ such that $\sqrt{(k+3)(k+4)} > \boldsymbol{\kappa} \frac{\pi}{2}$. Note that a higher value of *k* implies a higher degree of the downward Taylor approximations and of the polynomial $P(x)$ in (2) (for instance, see [10] and [12]).

Several more ideas to solve the above problem are proposed and considered below under the names of Methods A-D. In the following, the numbers $c_{k}$ and $d_{k}$ are those defined in Proposition 5.

### Method A

If $\delta < \frac{\pi}{2}$, find the smallest $k\in \mathbb{N}_{0}$ such that $d_{k}\in (\delta,\frac{\pi}{2} )$. Then $\widehat{k}=k$.

Note that Method A assumes solving a transcendental equation of the form $\cos {x}=\underline{T}_{ 4k+2}^{ \cos ,0}(x)$ that requires numerical methods.

### Method B

If $\delta < \frac{\pi}{2}$, find the smallest $k\in \mathbb{N}_{0}$ such that $c_{k}\in (\delta, \frac{\pi}{2} )$. Then $\widehat{k}=k$.

### Method C

If $\delta < \frac{\pi}{2}$, find the smallest $k\in \mathbb{N}_{0}$ such that $\underline{T}_{ 4k+2}^{ \cos,0}(\delta ) \geq 0$. Then $\widehat{k}=k$.

Note that Method B and Method C return the same output as for given *δ* and for every $k\in \mathbb {N}_{0}$ the following equivalence holds true:

$$\biggl( c_{k}\in \biggl( \delta , \frac{\pi }{2} \biggr) \wedge \underline{T}_{ 4k+2}^{ \cos ,0}(c_{k}) = 0 \biggr) \quad \Longleftrightarrow \quad \underline{T} _{ 4k+2}^{ \cos ,0}( \delta ) \geq 0. $$

As Method B assumes determining the root $c_{k}$ of the downward Taylor approximation $\underline{T}_{ 4k+2}^{ \cos ,0}(x)$ and Method C assumes checking the sign of the downward Taylor approximation at point $x=\delta $, it is notable that Method C presents a faster and simpler procedure.

### Method D

Eliminate all even degrees of the function cos*x* using the transformation

10 $$ \cos ^{2p}{ x} = \bigl( 1-\sin ^{2}{ x} \bigr) ^{p} = \sum_{i=0}^{p}(-1)^{i} \begin{pmatrix} p \\ i \end{pmatrix} \sin ^{2i}{x}. $$

Then $\widehat{k} = 0$.

Note that Method D can be applied for any $0<\delta \leq \pi /2$. Hence, if an MTP function $f(x)$ is considered in the whole interval $( 0, \frac{\pi}{2} )$, then Method D is applicable only (apart from the multiple-angle method). However, Method D implies an increase in the number of terms needed to be estimated. Let us represent a given MTP function *f* in the following form:

11 $$ f(x)=\sum_{i=1}^{\mathsf {m}} \alpha _{i}x^{p_{i}} \cos ^{2k_{i}} x\sin ^{r_{i}} x + f_{1}(x), $$

where there are no terms of the form $\cos^{2j}{ x}, j \in \mathbb{N}$, in $f_{1}(x)$. The elimination of all terms of the form $\cos ^{2k_{i}}{ x}$ from (11) using transformation (10) will increase the number of addends in (11), in the general case with $k_{1}+k_{2}+\cdots +k_{\mathsf {m}}$; consequently, it will increase the number of terms of the form $\sin ^{\ell}{ x}$, $\ell\in \mathbb{N}$, in (11) needed to be estimated.

### An algorithm based on the natural approach method

Let *f* be an MTP function and $\mathcal{I} \subseteq ( 0,\pi/2 )$. We concentrate on finding a polynomial $\mathcal{TP}^{f}(x)$ such that for every $x\in \mathcal{I}$,

$$f(x)>\mathcal{TP}^{f}(x). $$

In this case, the associated MTP inequality $f(x)>0$ can be proved if we show that for every $x\in \mathcal{I}$,

$$\mathcal{TP}^{f}(x)>0, $$

which is a decidable problem according to Tarski [22, 24]. The following algorithm describes the method for finding such a polynomial $\mathcal{TP}^{f}(x)$.


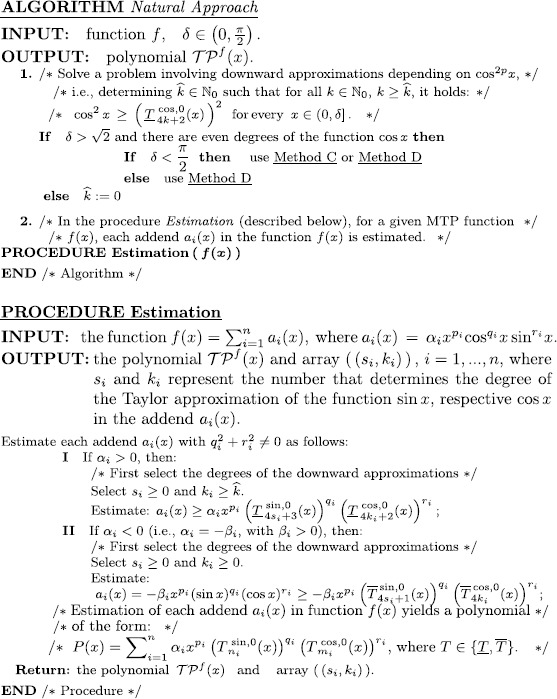


**Comment** on step II of the Procedure Estimation: in the general case, the addend $a_{i}(x)= -\beta _{i}x^{p_{i}}(\sin x)^{q_{i}}(\cos x)^{r_{i}}$ can be estimated in one of the following three ways:

(i) $a_{i}(x) =-\beta_{i}x^{p_{i}}(\sin x)^{q_{i}}(\cos x)^{r_{i}}\geq \beta_{i}x^{p_{i}} (\underline{T}^{\sin,0}_{4s_{i}+3}(x) )^{ q_{i}} (-\overline{T}^{\cos,0}_{4k_{i}}(x) )^{ r_{i}}$,
(ii) $a_{i}(x) =-\beta_{i}x^{p_{i}}(\sin x)^{q_{i}}(\cos x)^{r_{i}}\geq\beta_{i}x^{p_{i}} (-\overline{T}^{ \sin,0}_{4s_{i}+1}(x) )^{ q_{i}} (\underline{T}^{ \cos,0}_{4k_{i}+2}(x) )^{ r_{i}}$,
(iii) $a_{i}(x) =-\beta_{i}x^{p_{i}}(\sin x)^{q_{i}}(\cos x)^{r_{i}}\geq - \beta_{i}x^{p_{i}} (\overline{T}^{\sin,0}_{4s_{i}+1}(x) )^{ q_{i}} (\overline{T}^{ \cos,0}_{4k_{i}}(x) )^{ r_{i}}$.

Note that for fixed $s_{i}, k_{i}, q_{i} $ and $r_{i}$, the method (iii) generates polynomials of the smallest degree.

We present the following characteristic [28, 29] for the *Natural Approach* algorithm.

#### Theorem 8

*The Natural Approach algorithm is correct*.

#### Proof

Every step in the algorithm is based on the results obtained from Lemmas 1-4 and Proposition 5. Hence, for every input instance (i.e., for any MTP function $f(x)$ over a given interval ${\mathcal {I}}\subseteq (0, \pi/2 )$), the algorithm halts with the correct output (i.e., the algorithm returns the corresponding polynomial). □

## Some applications of the algorithm

We present an application of the *Natural Approach* algorithm in the proof (Application 1 - Theorem 9) of certain new rational (Padé) approximations of the function cos^2^ *x*, as well as in the improvement of a class of inequalities (20) by Yang (Application 2, Theorem 10).

### Application 1

Bercu [7] used the Padé approximations to prove certain inequalities for trigonometric functions. Let us denote by $( f(x) ) _{[m/n]}$ the Padé approximant $[ m/n ] $ of the function $f(x)$.

In this example we introduce a constraint of the function cos^2^ *x* by the following Padé approximations:

$$\bigl(\cos^{2}{ x} \bigr)_{[6/4]} = \frac{-59 x^{6} + 962 x^{4} - 3{,}675 x^{2} + 4{,}095}{17 x^{4} + 420 x^{2}+ 4{,}095} $$

and

$$\bigl(\cos^{2}{ x} \bigr)_{[4/4]} = \frac{163 x^{4} - 780 x^{2} + 945}{13 x^{4} + 165 x^{2} + 945}. $$

### Theorem 9

*The following inequalities hold true for every* $x \in ( 0, \frac{\pi}{2} )$:

12 $$ \bigl(\cos^{2}{ x} \bigr)_{[6/4]} < \cos ^{2}{ x} < \bigl(\cos^{2}{ x} \bigr)_{[4/4]}. $$

### Proof

We first prove the left-hand side inequality (11). Using the computer software for symbolic computations, we can conclude that the function $G_{1}(x) = ( \cos ^{2}{ x} ) _{[6/4]}$ has exactly one zero $\delta =1.551413\ldots$ in the interval $( 0, \frac{\pi}{2} )$. As $G_{1}(0)=1>0$ and $G_{1} ( \frac{\pi}{2} ) =-0.000431\ldots <0$, we deduce that

13 $$ G_{1}(x)\geq 0 \quad \mbox{for every } x\in (0,\delta ] $$

and

14 $$ G_{1}(x)< 0 \quad \mbox{for every } x\in \biggl(\delta , \frac{\pi}{2} \biggr). $$

Moreover, $G_{1}(x)<\cos ^{2}{ x}$ for every $x\in ( \delta , \frac{\pi}{2} ) $. We prove now that

15 $$ G_{1}(x)< \cos ^{2}{ x},\quad x\in ( 0, \delta ]. $$

We search a downward Taylor polynomial $\underline{T}_{ 4k+2}^{ \cos ,0}(x) $ such that for every $x\in ( 0, \delta ] $,

16 $$ G_{1}(x)< \bigl( \underline{T}_{ 4k+2}^{ \cos ,0}(x) \bigr) ^{2}< \cos ^{2}{ x}. $$

We apply the *Natural Approach* algorithm to the function $f(x)=\cos ^{2}{ x}$, $x\in (0,\delta ]$, to determine the downward Taylor polynomial $\underline{T}_{ 4k+2}^{ \cos ,0}(x)$ such that

$$\bigl( \underline{T}_{4k+2}^{ \cos ,0}(x) \bigr)^{2} < \cos ^{2} x,\quad x\in (0,\delta]. $$

We can use Method C or Method D from the *Natural Approach* algorithm since $\delta < \frac{\pi}{2}$. In this proof, we choose Method C.

The smallest *k* for which $\underline{T}_{ 4k+2}^{ \cos ,0}(\delta )>0$ is $k=1$. Therefore $\widehat{k}=1$. In the *Estimation* procedure only step I can be applied to the (single) addend cos^2^ *x*. In this step, $s_{1}\geq 0$ and $k_{1}\geq \widehat{k}=1$ should be selected. Let us select $s_{1}=0$ and $k_{1}=2$.^1^ As a result of this selection, the output of the *Natural Approach* algorithm is the polynomial

$$\mathcal{TP}(x)= \bigl( \underline{T}_{10}^{ \cos ,0}(x) \bigr) ^{2}= \biggl(1- \frac{x^{2}}{2!}+ \frac{x^{4}}{4!}- \frac{x^{6}}{6!}+ \frac{x^{8}}{8!}- \frac{x^{10}}{10!} \biggr)^{2}. $$

We prove that

17 $$ \bigl( \underline{T}_{10}^{ \cos ,0}(x) \bigr) ^{2}-G_{1}(x)>0,\quad x\in ( 0, \delta ] . $$

This is true since

$$\bigl( \underline{T}_{10}^{ \cos ,0}(x) \bigr) ^{2}-G_{1}(x)= \frac{x^{12}}{13{,}168{,}189{,}440{,}000 (17 x^{4}+420 x^{2}+4{,}095)} Q(x), $$

where

$$\begin{aligned} Q(x) = & 17 x^{12}+15 x^{8}\bigl(15{,}837-176 x^{2}\bigr)+8{,}100 x^{4}\bigl(64{,}519-1{,}687 x^{2}\bigr) \\ & {}+3{,}200 \bigl(50{,}205{,}015-4{,}035{,}906 x^{2}\bigr) > 0. \end{aligned}$$

Finally, we have $G_{1}(x)<\cos ^{2}{ x}$ for every $x\in ( 0, \delta ]$. According to (14), we have

$$G_{1}(x)< \cos ^{2}{ x} \quad \mbox{for every } x\in \biggl( 0, \frac{\pi}{2} \biggr). $$

Now we prove the right-hand side inequality (12). For $G_{2}(x)= ( \cos ^{2}{ x} ) _{[4/4]}$, we prove the following inequalities for every $x\in ( 0,\frac{\pi}{2} )$:

18 $$ \cos ^{2}{ x}< \bigl( \overline{T}_{8}^{ \cos ,0}(x) \bigr) ^{2}< G_{2}(x). $$

Based on Proposition 5, it is enough to prove that for every $x\in (0,\frac{\pi}{2} )$,

19 $$ \bigl( \overline{T}_{8}^{ \cos {x},0}(x) \bigr) ^{2}< G_{2}(x). $$

This is true as

$$G_{2}(x)- \bigl( \overline{T}_{8}^{ \cos {x},0}(x) \bigr) ^{2} = \frac{x^{10} }{1{,}625{,}702{,}400 (13 x^{4}+165 x^{2}+945)} R(x), $$

where

$$R(x)=x^{8}\bigl(1{,}291-13x^{2}\bigr)+x^{4} \bigl(2{,}004{,}240-66{,}913x^{2}\bigr)+480\bigl(632{,}604-74{,}625x^{2} \bigr) > 0. $$

Since $\cos ^{2}{ x}\leq ( \overline{T}_{4k}^{ \cos ,0}(x) ) ^{2} $, for every $k\in \mathbb {N}_{0}$ and all $x\in (0, \frac{\pi}{2})$, we have

$$\cos ^{2}{ x}< G_{2}(x)\quad\mbox{for every } x\in \biggl( 0, \frac{\pi}{2} \biggr) . $$

 □

### Note

Using Padé approximations, Bercu [7, 13] recently refined certain trigonometric inequalities over various intervals $\mathcal{I}=(0,\delta ) \subseteq (0, \frac{\pi}{2})$. All such inequalities can be proved in a similar way and using the *Natural Approach* algorithm as in the proof of Theorem 9.

### Application 2

Jang [6] proved the following inequalities for every $x\in ( 0,\pi )$:

20 $$ \cos ^{2}{ \frac{x}{2}}\leq \frac{\sin {x}}{x} \leq \cos ^{3}{ \frac{x}{3}}\leq \frac{2+\cos {x}}{3}. $$

Previously, Klén et al. [2] proved the above inequality on $(0,\sqrt{27/5})$ only.

In this example we propose the following improvement of (20).

### Theorem 10

*The following inequalities hold true for every* $x \in (0, \pi )$ *and* $a \in (1, \frac{3}{2} )$:

21 $$ \cos^{2}{ \frac{x}{2}} \leq \biggl( \frac{\sin{x}}{x} \biggr)^{a} \leq \frac{\sin{x}}{x}. $$

### Proof

As $a>1$ and $0< \frac{\sin {x}}{x}<1$, we have

$$\biggl( \frac{\sin {x}}{x} \biggr) ^{ a} < \frac{\sin {x}}{x}. $$

We prove now the following inequality:

22 $$ \cos ^{2}{ \frac{x}{2}}< \biggl( \frac{\sin {x}}{x} \biggr) ^{ a} $$

for every $x\in ( 0,\pi ) $ and $a\in ( 1, \frac{3}{2} ) $. It suffices to show that the following mixed logarithmic-trigonometric-polynomial function [11]

23 $$ F(x)=a\ln \biggl( \frac{\sin {x}}{x} \biggr) -2\ln \biggl( \cos { \frac{x}{2} } \biggr) $$

is positive for every $x\in ( 0,\pi ) $ and $a\in ( 1, \frac{3}{2} ) $. Given that

24 $$ \lim_{x\rightarrow 0}F(x)=0, $$

based on the ideas from [11], we connect the function $F(x)$ to the analysis of its derivative

$$F^{\prime }(x)= \frac{1}{2} \frac{ f (\frac{x}{2} )}{x\sin \frac{x}{2}\cos \frac{x}{2}}, $$

where

25 $$ f(t)=4t(a-1)\cos ^{2}{ t}-2a\sin {t}\cos {t}-2t(a-2). $$

Let us note that $F^{\prime }(x)$ is the quotient of two MTP functions.

The inequality $F^{\prime }(x)>0$ is equivalent to $f(t)>0$. The proof of the later inequality will be done using the *Natural Approach* algorithm for the function $f(t)$ on $( 0, \frac{\pi}{2} ) $, with $a \in ( 1, \frac{3}{2} )$. As before, we search a polynomial $\mathcal{TP}(t)$ such that

$$f(t)>\mathcal{TP}(t)>0. $$

In step 1 of the *Natural Approach* algorithm, we can use Method D only because $\delta = \frac{\pi}{2}$. Then

26 $$\begin{aligned} f(t) = & 4 t (a-1) \bigl(1-\sin ^{2}{t}\bigr) - 2 a \sin {t} \cos {t} - 2 t (a-2) \\ = & 4 t (1-a) \sin ^{2}{t} - 2 a \sin {t} \cos {t} + 2 t a \end{aligned}$$

with $\widehat{k} = 0$. In the *Estimation* procedure only^2^ step II can be applied to the first and second addends in (26), where $s_{i}\geq 0$ and $k_{i}\geq 0$, $i=1,2$, should be selected. Let us, for example, select $s_{1}=k_{1}=s_{2}=k_{2}=1$. As a result of this selection, the *Natural Approach* algorithm yields the polynomial

$$\mathcal{TP}(t) = 4t(1 - a) \biggl( t - \frac{1}{6} t^{3} + \frac{1}{120} t^{5} \biggr)^{ 2} - 2a \biggl( t - \frac{1}{6} t^{3} + \frac{1}{120} t^{5} \biggr) \biggl( 1 - \frac{1}{2} t^{2} + \frac{1}{24} t^{4} \biggr) + 2 t a $$

for which $f(t)>\mathcal{TP}(t)$, for every $t \in ( 0, \frac{\pi}{2} )$ and $a \in ( 1, \frac{3}{2} ) $. The inequality $f(t)>0$ is reduced to a decidable problem

27 $$ \mathcal{TP}(t)>0, \quad \mbox{for every } t\in \biggl( 0, \frac{\pi}{2} \biggr) \mbox{ and } a\in \biggl( 0,\frac{3}{2} \biggr) . $$

The sign of the polynomial $\mathcal{TP}(t)$ can be determined in several ways. For example, let us represent the polynomial $\mathcal{TP}(t)$ as

28 $$ \mathcal{TP}(t) = p(t)a + q(t), $$

where

$$p(t) = - \frac{t^{3} ( 2t^{8} - 75t^{6} + 1{,}120t^{4} - 7{,}680t^{2} + 19{,}200 ) }{7{,}200} $$

and

$$q(t) = 4 t \biggl( t - \frac{1}{6} t^{3} + \frac{1}{120} t^{5}\biggr)^{ 2} . $$

For every fixed $t\in ( 0,\frac{\pi}{2} )$, the function $\mathcal{TP}(t)=p(t)a+q(t)$ is linear, monotonically decreasing with respect to $a \in (1, \frac{3}{2} )$ since for every $t \in ( 0, \frac{\pi}{2} )$,

$$p(t)=- \frac{t^{3}}{7{,}200} \bigl(2 t^{8}+5 t^{4} \bigl(224-15t^{2}\bigr)+3{,}840 \bigl(5-2t^{2}\bigr) \bigr) < 0. $$

Hence, for every fixed $t \in ( 0, \frac{\pi}{2} )$, the value of (28) is greater than the value of the same expression for $a= \frac{3}{2}$:

$$p(t) \frac{3}{2} + q(t)=- \frac{t^{5}}{14{,}400} \bigl(2 t^{6}-65 t^{4}+800 t^{2}-3{,}840\bigr). $$

But

$$p(t) \frac{3}{2} + q(t)= \frac{t^{5}}{14{,}400} \bigl( t^{4} \bigl(65-2 t^{2}\bigr) +160\bigl( 24-5t^{2}\bigr) \bigr)>0,$$

so inequality (27) is true; and consequently, $F^{\prime }(x)>0$ on $(0,\pi )$ for every $a\in ( 1, \frac{3}{2} ) $. But $\lim_{x\rightarrow 0}{F(x)}=0$, so $F(x)>0$ on $(0,\pi)$ for every $a\in ( 1,\frac{3}{2} )$. □

### Remark on Theorem 10

Let us consider possible refinements of inequality (20) by a real analytical function $\varphi _{a}(x) = ( \frac{\sin x}{x} ) ^{ a}$ for $x \in ( 0,\delta ) $ and $a \in \mathbb{R}$. The function $\varphi _{a}(x)$ is real analytical as it is related to the analytical function

29 $$ t(x)=a\ln \biggl( \frac{\sin x}{x} \biggr) =a \sum _{k=1}^{\infty } \frac{(-1)^{k}2^{2k-1}B_{2k}}{k(2k)!} x^{2k} $$

($B_{i}$ are the Bernoulli numbers; see, e.g., [30]). The following consideration of the sign of the analytical function in the left and right neighborhood of zero is based on Theorem 2.5 from [8]. Let us consider the real analytical function

30 $$ f_{1}(x) = \biggl( \frac{\sin x}{x} \biggr) ^{ a}-\cos ^{2}{ \frac{x}{2}} = \biggl( - \frac{a}{6}+ \frac{1}{4} \biggr)x^{2} + \biggl( \frac{{a}^{2}}{72} - \frac{a}{180} - \frac{1}{48} \biggr){x}^{4} + \cdots, $$

$x \in (0,\pi)$. The restriction

31 $$ f_{1}^{\prime \prime }(0)=- \frac{a}{3}+ \frac{1}{2} >0, $$

i.e.,

32 $$ a\in \biggl( -\infty , \frac{3}{2} \biggr), $$

is a necessary and sufficient condition for $f_{1}(x)>0$ to hold on an interval $(0,\delta _{1}^{(a)} )$ (for some $\delta _{1}^{(a)} > 0$). Also, the restriction

33 $$ a\in \biggl( \frac{3}{2},\infty \biggr) $$

is a necessary and sufficient condition for $f_{1}(x)<0$ to hold on an interval $(0,\delta _{2}^{(a)})$ (for some $\delta _{2}^{(a)} > 0$). The following equivalences hold true for every $x \in (0,\pi )$:

34 $$\begin{aligned}& a\in ( 1,\infty ) \quad \Longleftrightarrow \quad \biggl( \frac{\sin x}{x} \biggr) ^{ a}< \frac{\sin x}{x}, \end{aligned}$$

35 $$\begin{aligned}& a \in ( -\infty ,1 ) \quad \Longleftrightarrow \quad \frac{\sin x}{x}< \biggl( \frac{\sin x}{x} \biggr) ^{ a}. \end{aligned}$$

The refinement in Theorem 10 is given based on the possible values of the parameter *a* in (33) and (34). A similar analysis shows us that only the following refinements of inequality (20) are possible.

### Corollary 11

*Let* $a\in [\frac{3}{2}, +\infty )$. *There exists* $\delta >0$ *such that for every* $x\in ( 0,\delta ) $, *it holds*

36 $$ \biggl( \frac{\sin {x}}{x} \biggr) ^{a}\leq \cos ^{2} \frac{x}{2}. $$

### Corollary 12

*Let* $a\in ( -\infty ,1 ) $. *There exists* $\delta >0$ *such that for every* $x\in ( 0,\delta ) $, *it holds*

37 $$ \frac{2+\cos {x}}{3}\leq \biggl( \frac{\sin {x}}{x} \biggr) ^{a}. $$

## Conclusions and future work

The results of our analysis could be implemented by means of an automated proof assistant [31], so our work is a contribution to the library of automatic support tools [32] for proving various analytic inequalities.

Our general algorithm associated with the natural approach method can be successfully applied to prove a wide category of classical MTP inequalities. For example, the *Natural Approach* algorithm has recently been used to prove several open problems that involve MTP inequalities (see, e.g., [8–12]).

It is our contention that the *Natural Approach* algorithm can be used to introduce and solve other new similar results. Chen [4] used a similar method to prove the following inequalities, for every $x\in ( 0,1 )$:

$$2+\frac{17}{45}x^{3}\arctan x< \biggl( \frac{\arcsin x}{x} \biggr)^{ 2} + \frac{\arctan x}{x} $$

and

$$2+\frac{7}{20}x^{3}\arctan x< 2 \biggl( \frac{\arcsin x}{x} \biggr) + \frac{\arctan x}{x}; $$

then he proposed the following inequalities as a conjecture:

$$\biggl( \frac{\arcsin x}{x} \biggr)^{ 2} + \frac{\arctan x}{x} < 2+ \frac{\pi ^{2}+\pi -8}{\pi }x^{3}\arctan x, \quad x \in (0,1 ) $$

and

$$2 \biggl( \frac{\arcsin x}{x} \biggr) + \frac{\arctan x}{x} < 3 + \frac{5 \pi-12}{\pi} x^{3} \arctan x, \quad x \in (0,1 ). $$

Very recently, Malešević et al. [12] solved this open problem using the same procedure, i.e., the natural approach method, associated with upwards and downwards approximations of the inverse trigonometric functions.

Finally, we present other ways for approximating the function $\cos ^{2n}{ x}$, $n\in \mathbb {N}$. It is well known that the power series of the function $\cos ^{2n}{ x}$ converges to the function everywhere on $\mathbb {R}$. The power series of the function $\cos ^{2n}{ x}$ is an alternating sign series. For example, for $n=1$ and $x\in\mathbb{R}$, we have

$$\cos ^{2}{ x}=1-x^{2}+\frac{1}{3}x^{4}- \frac{2}{45}x^{6}+\cdots =1+ \sum_{k=0}^{\infty }{ \frac{2^{2k-1}(-1)^{k}}{(2k)!}x^{2k}}. $$

Therefore, for the above power (Taylor) series, it is not hard to determine (depending on *m*) which partial sums (i.e., Taylor polynomials) $T_{m}^{ \cos ^{2} x, 0} (x)$ become good downward or upward approximations of the function cos^2^ *x* in a given interval ${\mathcal {I}}$. Assuming the following representation of the function $\cos ^{2n}{ x}$ in power (Taylor) series

$$\cos ^{2n}{ x} =a_{0}^{(2n)}-a_{2}^{(2n)}x^{2}+a_{4}^{(2n)}x^{4}-a_{6}^{(2n)}x^{6}+ \cdots, $$

with $a_{j}^{(2n)}>0$ $( j=0,2,4,6,\ldots ) $, the power (Taylor) series of function $\cos ^{2n+2}{ x}$ will be an alternating sign series as follows:

$$\begin{aligned} \cos ^{2n+2}{ x} = & \cos ^{2}{ x}\cdot \cos ^{2n}{ x} \\ = & \underbrace{a_{0}^{(2n)}}_{a_{0}^{(2n+2)}} \\ & {}-\underbrace{\bigl(a_{0}^{(2n)}+a_{2}^{(2n)} \bigr)} _{a_{2}^{(2n+2)}}x^{2} \\ & {}+\underbrace{\biggl( \frac{1}{3} a_{0}^{(2n)}+a_{2}^{(2n)}+a_{4}^{(2n)} \biggr)} _{a_{4}^{(2n+2)}}x^{4} \\ & {}-\underbrace{\biggl( \frac{2}{45} a_{0}^{(2n)} + \frac{1}{3} a_{2}^{(2n)} + a_{4}^{(2n)} + a_{6}^{(2n)} \biggr)}_{a_{6}^{(2n+2)}}x^{6} \\ & {}+ \cdots \end{aligned}$$

with $a_{j}^{(2n+2)}>0$ ($j=0,2,4,6,\ldots$).

Therefore, in general, for the function $\cos^{2n}{ x}$, it is possible to determine, depending on the form of the real natural number *m*, the upward (downward) Taylor approximations $\overline{T}^{ \cos^{2n}{x}, 0}_{m}(x)$ ($\underline{T}^{ \cos^{2n} x,0}_{m}(x)$) that are all above (below) the considered function in a given interval $\mathcal{I}$. Such estimation of the function $\cos^{2n}{x}$ and the use of corresponding Taylor approximations will be the object of future research.

## Footnotes

1 For the selection $s_{1}=0$ and $k_{1}=1$, the output of the *Natural Approach* algorithm is the polynomial

$$\mathcal{TP}(x)=\underline{T}_{6}^{ \cos ,0}(x)= 1- \frac{x^{2}}{2!}+ \frac{x^{4}}{4!}- \frac{x^{6}}{6!} $$

such that $\mathcal{TP}(x) \lessgtr G_{1}(x)$ holds for some $x\in (0,\delta ]$.

2 Because for every fixed $a\in ( 1,\frac{3}{2} ) $: $\alpha _{1}=4(1-a)<0$ and $\alpha _{2}=-2a<0$.
